# Increase in length of stay and overcost attributable to hospital-acquired infections in a pediatric unit in Senegal

**DOI:** 10.1186/1753-6561-5-S6-P249

**Published:** 2011-06-29

**Authors:** A Ndir, A Cisse, LP Nadiele, NM Dia Badiane, B Ndoye

**Affiliations:** 1 Pronalin, Dakar, Senegal; 2 Des, Ministere Sante, Dakar, Senegal; 3 HPD, Dakar, Senegal; 4 CHNU Fann, Dakar, Senegal

## Introduction / objectives

Economic data on healthcare–associated infections (HCAI) in resource-poor countries are practically nonexistent despite the importance of this issue. The objective of our study was to estimate the increase in length of stays and the overcost attributable to HCAI in a pediatric unit in a senegalese hospital to show their economic impact.

## Methods

Retrospective analysis on hospital stays' database in the pediatric unit at Hopital Principal de Dakar between September and November 2010. Patients with a positive bacteriological test were selected. Probable cases of HCAI were included on clinical and bacteriological data (presence of multiresistant bacteria). Cases were matched to control patients (with no infection) by sex, age and hospitalization period. The overcost is the difference cost of cases' stay and control patient' stay.

## Results

2 cases were excluded from the analysis because of death. 10 cases of HCAI were found in 9 patients aged between 0 an 5 years. Among them, 6 were bloodstream infections and 1 was an urinary infection. The increase in hospital stay potentially attributable to bloodstream infections was of 13 days with a mean overcost of 1203€.

## Conclusion

It seems necessary to carry out this case-control study on more patients and to take into account the reason of hospitalization to increase the strength of the results. However, our analysis showed the economic burden of HCAI which is considerable in resource-poor countries where healthcare budget are insufficient. These results should prompt authorities to invest substantially in HCAI prevention.

## Disclosure of interest

None declared.

## Note

Abstract also presented as P350.

